# Protein Kinase Inhibitors CK59 and CID755673 Alter Primary Human NK Cell Effector Functions

**DOI:** 10.3389/fimmu.2013.00066

**Published:** 2013-03-18

**Authors:** Maxi Scheiter, Björn Bulitta, Marco van Ham, Frank Klawonn, Sebastian König, Lothar Jänsch

**Affiliations:** ^1^Research Group Cellular Proteomics, Helmholtz Centre for Infection ResearchBraunschweig, Germany; ^2^Department of Computer Science, Ostfalia University of Applied SciencesWolfenbuettel, Germany

**Keywords:** NK cells, immune modulation, signaling pathways, PKD, CaMKII, effector function, Fyn, CID755673

## Abstract

Natural killer (NK) cells are part of the innate immune response and play a crucial role in the defense against tumors and virus-infected cells. Their effector functions include the specific killing of target cells, as well as the modulation of other immune cells by cytokine release. Kinases constitute a relevant part in signaling, are prime targets in drug research and the protein kinase inhibitor Dasatinib is already used for immune-modulatory therapies. In this study, we tested the effects of the kinase inhibitors CK59 and CID755673. These inhibitors are directed against calmodulin kinase II (CaMKII; CK59) and PKD family kinases (CID755673) that were previously suggested as novel components of NK activation pathways. Here, we use a multi-parameter, FACS-based assay to validate the influence of CK59 and CID755673 on the effector functions of primary NK cells. Treatment with CK59 and CID755673 indeed resulted in a significant dose-dependent reduction of NK cell degranulation markers and cytokine release in freshly isolated Peripheral blood mononuclear cell populations from healthy blood donors. These results underline the importance of CaMKII for NK cell signaling and suggest protein kinase D2 as a novel signaling component in NK cell activation. Notably, kinase inhibition studies on pure NK cell populations indicate significant donor variations.

## Introduction

Natural killer (NK) cells establish the first line of innate immune defense against pathogen-infected cells and tumor cells, as well as a certain set of hematopoietic cells (Vivier et al., 2008; Bryceson et al., 2009), a process also known as natural cytotoxicity.

Natural cytotoxicity encompasses coordination of following steps: contact with target cells, adhesion, synapse formation, granule polarization, and granule exocytosis. These steps are mainly under the control of a multiplicity of germline-encoded activating and inhibitory surface receptors, which bind ligands expressed on target cells (Bryceson et al., 2006, 2011; Lanier, 2008; Pegram et al., 2010). In addition to natural cytotoxicity, NK cells mediate antibody-dependent cellular cytotoxicity (ADCC). ADCC depends on FcγRIIIA receptor expression (CD16), so that cells can become targets that do normally not activate NK effector responses (Forthal et al., 1999). In contrast, natural cytotoxicity occurs independently of antigen recognition and is mainly studied *in vitro* by using cell lines like K562 (Hanson et al., 2007).

K562 target cells express very low amounts of MHC class I (missing self). However, natural cytotoxicity depends not only on an absent inhibitory signal, but also on activating signals that are necessary for NK activation and tumor cell lysis (Moretta et al., 2000). Hence, K562 cells express ligands that bind activating NK cell receptors, e.g., ULBP2 and MICA/B, the ligands of NKG2D (Li et al., 2008), B7–H6 as the ligand of NKp30 (Brandt et al., 2009) and Nectin-2, which acts as a ligand for DNAM-1 (Moretta et al., 2000). K562 do not express CD48 (the 2B4 NK receptor ligand), as well as classical (HLA-A, B, C) and non-classical (HLA-E) HLA class I molecules (Hanson et al., 2007). Additionally, natural cytotoxicity leads to the secretion of pro-inflammatory cytokines like TNF-α and IFN-γ (Vivier et al., 2008) and can be further triggered by supplementing interleukins like IL-2, IL-12, IL-18, as well as IFN-γ.

After receptor engagement, protein kinases, like Protein Kinase C-θ (PKCθ), Phophatidyl-inositol-3-OH kinase (PI3K) or Src family kinases (SFKs) like FYN, induce signaling networks controlling NK cell effector functions (Brumbaugh et al., 1997; Kerr and Colucci, 2011; Merino et al., 2012). ADCC- and natural cytotoxicity-induced signal transduction pathways share many signaling components and a kind of core signaling network was suggested (König et al., 2012). The same study described post-translational responses of kinases following NK cell activation indicating their role in proximal signaling pathways. Among 188 kinases that were characterized by accurate mass spectrometry in IL-2-expanded human NK cells, an increased phosphorylation of FYN, the Calcium/Calmodulin Kinase II (CaMKII) and Protein Kinase D2 (PKD2), was reproducibly observed after receptor engagement (König et al., 2012). Nevertheless, our knowledge about the signaling controlling ADCC/natural cytotoxicity is very fragmentary to this date.

Modulation of immune responses is a general therapeutic strategy. Up to now, NK cell based therapies against cancer are performed by using IL-2 or other antibody-based therapies (Vivier et al., 2012). Furthermore, clinically relevant kinase inhibitors were recognized to cause significant immune-modulatory effects. Studies on NK cells were conducted by using kinase inhibitors, like Imatinib and Nilotinib, both specifically targeting BCR/ABL, PDGFR, and c-KIT, as well as on Dasatinib, which is additionally directed against the Src kinase family. These studies confirmed their direct inhibitory effects on NK cell effector functions (Krieg and Ullrich, 2012).

In the case of Dasatinib, a direct inhibition of NK cell effector functions resulted from its effects on PI-3 kinase and ERK1/2 signaling cascades (Salih et al., 2010).

The protein kinase CaMKII was previously described to play an important role in NK cell activation, after being induced by lymphocytes function-associated antigen 1 (LFA-1). Adding the CaMKII inhibitors KN62/KN93 reduced the secretion of lytic granules and the cytotoxic activity remarkably in CD3^−^CD16^+^ NK cells. Furthermore, it was shown that the HIV-1 Tat protein is able to block calcium influx and impairs CaMKII induction, which points to a clinical relevance of the CaMKII kinase (Poggi et al., 2002).

The PKD kinase family has been implicated in a variety of cellular processes, including cell proliferation, cell survival (Storz et al., 2003), gene expression (Ha et al., 2008), protein trafficking (Bankaitis, 2002), cell motility (Prigozhina and Waterman-Storer, 2004), and immune responses (Matthews et al., 2012). PKD kinases are frequently de-regulated in cancer, become of interest in tumor biology and are considered as therapeutic targets for drug development and applications in immunotherapy (Chen et al., 2008).

In this study and beside of Dasatinib, the next generation CaMKII inhibitor CK59, which specifically binds CaMKII-β and CaMKII-δ (Konstantopoulos et al., 2007) and the PKD family directed kinase inhibitor CID755673 (Sharlow et al., 2008; George et al., 2011) were used to determine their potential effects on NK cell effector functions. Peripheral blood mononuclear cells (PBMCs), isolated from human blood donations, were pre-treated with various doses of these drugs and degranulation, TNF-α and IFN-γ secretion were analyzed by multicolor FACS measurements. In addition to the results of Salih et al. (2010), we observed remarkably reduced TNF-α release and degranulation events after Dasatinib treatment. Application of the further improved CK59 (CaMKII-directed) inhibitor led to strongly abrogated NK cell degranulation and cytokine release, but an even more pronounced reduction of NK cell effector functions was observed after using CID755673. Additionally, this study revealed significant donor variations at the level of primary NK cells.

## Materials and Methods

### Human subjects and cell lines

Buffy coats from the blood of healthy human volunteers were obtained from the Institute for Clinical Transfusion Medicine, Klinikum Braunschweig, Germany. Leukocyte-depleted peripheral blood from regular donations was anonymized in accordance with the guidelines of the Declaration of Helsinki. PBMCs were isolated from buffy coats by Biocoll density gradient centrifugation (Biochrome AG). PBMCs were directly used for experiments or cultured in complete RPMI 1640 medium (GIBCO) supplemented with 10% fetal bovine serum (FBS) gold (PAA Laboratories), 2 mM l-glutamine, 50 units/ml penicillin and 50 μg/ml streptomycin (all Invitrogen). The human erythroleukemia cell line K562 (kindly provided by Dr. P. Riese – Department of Vaccinology; Helmholtz Centre for Infection Research, Braunschweig) was maintained in RPMI 1640 (Gibco) supplemented with 10% FBS gold (PAA Laboratories), 50 units/ml penicillin, and 50 μg/ml streptomycin (all Invitrogen). NK cells were isolated from PBMCs using the NK cell isolation kit (Miltenyi) according to the manual. Isolated NK cells were incubated in IMDM (Gibco), supplemented with 10% human serum, 2 mM l-glutamine, 50 units/ml penicillin and 50 μg/ml streptomycin, 1% Sodium Pyruvate, and 1% NEAA (all Invitrogen).

### Antibodies and fluorescent reagents

The following fluorochrome-conjugated monoclonal antibodies (mAbs) were used for flow cytometry: anti-CD3 (BW264/56, Miltenyi), anti-CD56 (AF12-7H3, Miltenyi), anti-CD107a (H4A3; BioLegend); anti-CD14 (MΦP9, BD Bioscience), anti-CD19 (SJ25C1, BD Bioscience), anti-TNF-α (MAb11; eBioscience), and anti-IFN-γ (25723, BD Bioscience). A fixable cell viability cell dye (LIVE/DEAD near IR Cell Stain; 405 nm Invitrogen) was used to exclude dead cells from the analysis.

### Degranulation and cytokine release assay

Degranulation events and cytokine release were assessed as described previously (Bryceson et al., 2010). In brief, resting PBMCs or pure NK cells were incubated for 15 min at 37°C, 7.5% CO_2_ with the indicated dilutions of the kinase inhibitors (CK59-Merck; CID755673-TOCRIS BIOSCIENCE; Dasatinib-LC Laboratories) in complete RPMI 1640 medium. Cells were mixed with their natural target cells, K562 in V-bottomed 96-well plates at a ratio of 10:1 for assessing TNF-α and IFN-γ release and in a ratio of 1:1 for NK cell degranulation assays. IL-12 (10 ng/ml) and IL-18 (100 ng/ml; both from PeproTech) were added to determine cell viability and responsiveness as indicated (Figure A2 in Appendix). Cells were centrifuged at 30 × *g* for 3 min and incubated for 1 h at 37°C and 7.5% CO_2_. Thereafter, GolgiPlug and GolgiStop (both BD Bioscience) were added and cells were incubated for further 2 h to assess NK cell degranulation and for another 5 h to analyze cytokine release. Effector cells treated with DMSO served as negative controls. After incubation, cells were harvested by centrifugation (450 × *g*, 3 min) and surface stained (CD3, CD56, CD107a, CD14, CD19). After fixation (2% PFA) and permeabilization (0.5% Saponin in PBS) cells were intracellularly stained with fluorochrome-conjugated mAbs against IFN-γ and TNF-α.

### Flow cytometric analysis

Analysis of degranulation and cytokine release was carried out on a BD LSR II Flow Cytometer (BD Biosciences), using the FACSDiva (BD Biosciences) software for compensation. Data analysis was performed by using FlowJo version 7.6.5 (Treestar).

### Statistical analysis

In each experiment we used at least five human blood donors, from which two technical replicates were generated that defined groups for further statistical analyses. Comparison of inhibitor-treated groups to their corresponding DMSO control group was performed by Wilcoxon test (*P < 0.05, **P < 0.01, ***P < 0.001). Statistical analysis was performed by using R, version 2.15.1.

## Results

### Dasatinib, CK59, and CID755673 decrease NK cell degranulation and cytokine release in primary human PBMCs

**Figure A1:**
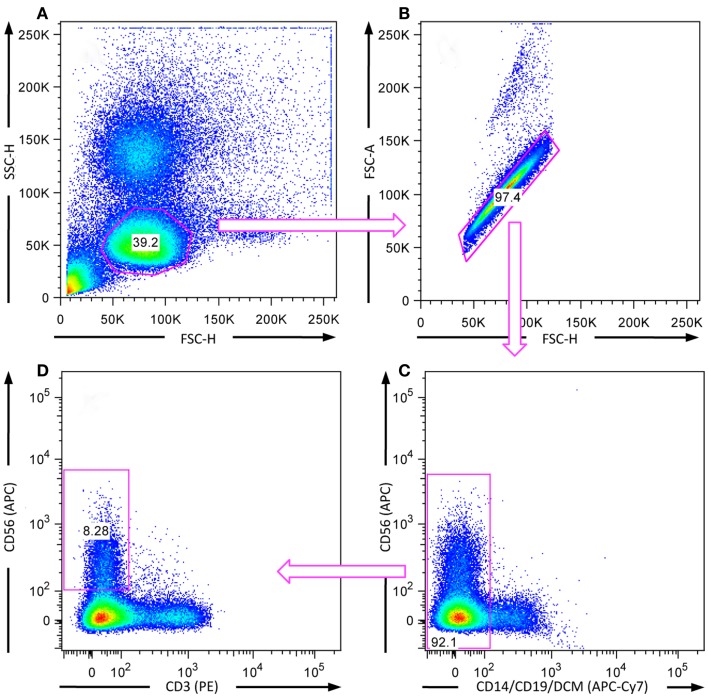
**Gating strategy to study effector functions of human CD3^−^CD56^+^ NK cells**. NK cells were co-incubated with K562 target cells for 6 h for assessment of cytokine production and 2 h for investigating degranulation at 37°C. After stimulation, cells were surface stained with fluorochrome-conjugated mAbs directed against CD3, CD14, CD19, CD56, and CD107a, as well as with DCM. The depicted gates were used to exclude **(A)** non-lymphocytes, **(B)** doublets, **(C)** CD14^+^/CD19^+^, and dead cells as well as **(D)** CD3^+^CD56^−^ cells from the analysis.

**Figure A2:**
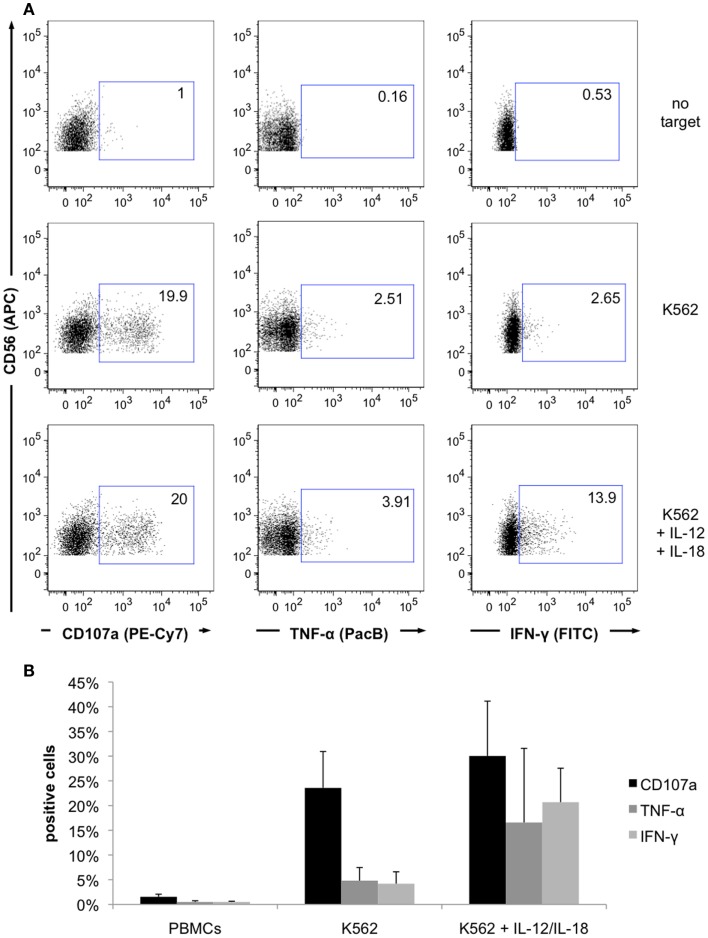
**Activation of NK cell effector functions by co-incubation with K562 target cells, IL-12 and IL-18**. **(A)** PBMCs were incubated with or without K562 target cells, as well as in combination with IL-12 and IL-18 for 6 h at 37°C, fixed, permeabilized, and stained with respective fluorochrome-conjugated mAbs. Dot plots show CD107a expression and cytokine production of NK cells without target cells or co-cultured with K562 cells, IL-12, and IL-18. Non-activated cells showed no significant responses (upper panel), incubation with target cells triggered degranulation and cytokine production (middle panel) and supplementation of IL-12 and IL-18 lead to higher cytokine production (lower panel). Results from one representative donor are shown. **(B)** Increase of NK cell degranulation and cytokine release by K562 target cells and supplementation of IL-12 and IL-18 to validate the viability and responsiveness of the cells. Values represent mean of five individual donors ±SD.

To assess the influence of Dasatinib (inhibits SFKs), CK59 (inhibits CaMKII), and CID755673 (inhibits PKD kinase family) on K562-induced natural cytotoxicity of NK cells, the effector functions (degranulation and cytokine release) were assessed by FACS-based methods as previously described (Bryceson et al., 2010). For these experiments PBMCs were isolated by density gradient centrifugation from buffy coats of healthy human blood donors. CD3^−^CD56^+^ NK cell populations were then defined by gating PBMCs on lymphocytes, excluding doublets, as well as excluding CD14^+^, CD19^+^, and dead cells (Figure A1 in Appendix). K562 cancer cells were used as target cells for the activation of NK cells and to study natural cytotoxicity within the PBMC fraction. IL-12 and IL-18 support NK cell cytokine release triggered by target cell recognition (Fauriat et al., 2010). By adding IL-12 and IL-18 the responsiveness and viability of the cells was tested. Co-stimulation with target cells, IL-12 and IL-18 significantly increased NK cell cytokine production and to a minor extent NK cell degranulation (Figure A2 in Appendix).

The specific inhibitors were added to PBMCs at indicated concentrations 15 min prior to activation with target cells. After 2 h of co-incubation with target cells, degranulation was analyzed by measuring CD107a surface expression. Cytokine release was determined by intracellular staining after treatment with GolgiPlug and GolgiStop (both BD Biosciences). As the inhibitors were delivered in DMSO, corresponding DMSO concentrations were added to control samples and data are presented as activated cells relative to those DMSO controls.

**Figure 1:**
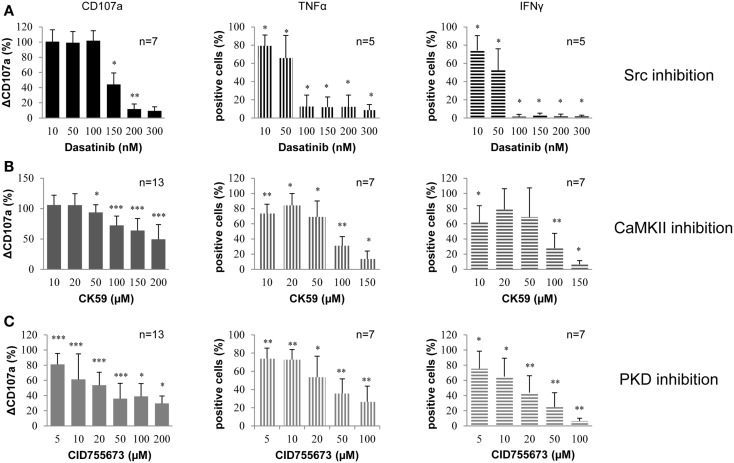
**Small molecule kinase inhibitors (Dasatinib, CK59, and CID755673) decrease NK cell degranulation and cytokine release in primary human PBMCs**. PBMCs were treated with **(A)** Dasatinib, directed against Src family kinases; **(B)** CK59, targeting CaMKII; and **(C)** CID755673, inhibiting PKD family kinases and subsequently incubated with K562 target cells at 37°C for 2 (degranulation) and 6 (cytokine release) hours, respectively. Cells were mixed with respective fluorochrome-conjugated mAbs and the frequency of degranulating or cytokine producing NK cells was determined *via* flow cytometry. Data represent values relative to corresponding DMSO control. Comparison of every inhibitor-treated group to its corresponding DMSO control group was performed by Wilcoxon test (*P < 0.05,**P < 0.01,***P < 0.001). Values represent mean of the indicated number of donors ±SD.

By measuring CD107a surface expression, it could be shown that 150 nM Dasatinib reduced degranulation by 50% and nearly led to complete abrogation at higher concentrations (Figure 1A). Dasatinib applied at concentrations lower than 150 nM had no significant effect on degranulation. Furthermore, Dasatinib led to a significant inhibition of NK cell TNF-α and IFN-γ production induced by target cell recognition, even at low concentrations. At concentrations equal to or higher than 100 nM, release of TNF-α was reduced to 15% while production of IFN-γ was almost abrogated.

Under the same conditions, CK59 inhibited NK cell degranulation also dose-dependently and more gradually in comparison to Dasatinib (Figure 1B). The kinase inhibitor CK59 also reduced the number of cytokine producing cells without displaying the same dose-dependence. Low inhibitor concentrations (10–50 μM) showed only a moderate reduction, whereas CK59 applied at higher concentrations strongly reduced NK cell cytokine production, which was nearly abrogated at 150 μM. In general, the effect of CK59 on TNF-α and IFN-γ production was comparable. Thus, CK59 led to dose-dependent inhibition of NK cell degranulation and had an even stronger effect on cytokine production at higher concentrations.

CID755673 reduced NK cell degranulation as well as NK cell cytokine release (Figure 1C). The number of degranulating cells was significantly reduced at an inhibitor concentration of 5 μM. At higher concentrations NK cell degranulation was reduced to 40%. Notably, it did not decrease further when concentrations of CID755673 were raised to more than 50 μM. However, increasing the concentration of the inhibitor to 100 μM led to a further reduction of cytokine production. TNF-α production was also decreased dose-dependently and the percentage of TNF-α-producing cells was reduced to approximately 25% at 100 μM CID755673. The production of IFN-γ was also decreased in a dose-dependent manner and almost completely abrogated at a concentration of 100 μM. In summary, CID755673 led to a significant inhibition of NK cell effector functions, in which IFN-γ responses could be nearly abolished.

Taken together, treatment of PBMC cultures with Dasatinib, CK59, and CID755673 resulted in a significant inhibition of NK cell effector functions, including degranulation and cytokine release.

### Donor-specific responses of pure NK cells treated with Dasatinib, CK59, and CID755673

To validate the effects of Dasatinib, CK59, and CID755673 on NK cells and to reveal the potential contribution of other PBMCs, NK cells were purified by MACS before inhibitor application and subsequent activation with K562 target cells. Stimulation and FACS analysis were performed as described before. Notably, donor variations became more obvious at the level of pure NK cells in contrast to PBMCs for all three inhibitors.

**Figure 2:**
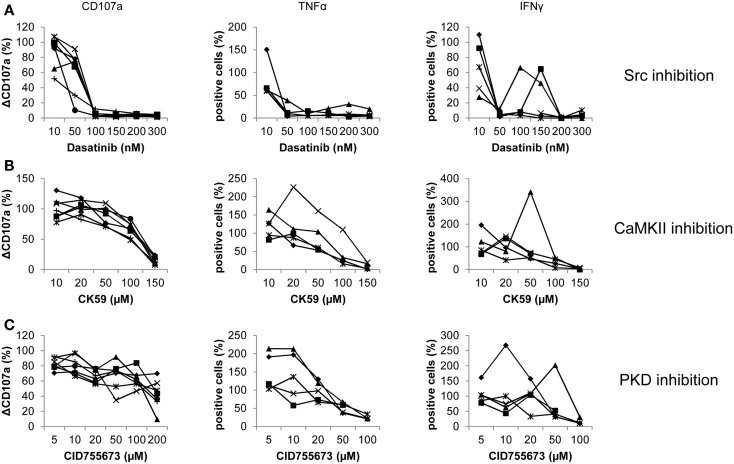
**Donor-specific responses of pure NK cells treated with Dasatinib, CK59, and CID755673**. Purified NK cells were treated with **(A)** Dasatinib, directed against Src kinases; **(B)** CK59, targeting CaMKII; and **(C)** CID755673, inhibiting PKD family kinases and subsequently incubated with K562 target cells at 37°C for 2 (degranulation) and 6 (cytokine release) hours, respectively. Cells were mixed with respective fluorochrome-conjugated mAbs and the frequency of degranulating or cytokine producing NK cells was determined *via* flow cytometry. Data represents values relative to corresponding DMSO control. Comparison of every inhibitor-treated group to its corresponding DMSO control group was performed by Wilcoxon test (*P < 0.05,**P < 0.01,***P < 0.001). Values represent mean of the indicated number of donors ±SD.

Dasatinib showed an even more pronounced effect on degranulation, which was completely abrogated at 100 nM (Figure 2A). Release of TNF-α and IFN-γ also showed a strong reduction at concentrations of 50 nM. Some donors exhibited an increased release of TNF-α and IFN-γ after an initial reduction. After nearly complete abrogation of TNF-α release, one donor showed an increase in cells positive for TNF-α peaking at 200 nM and 30% positive cells. Another donor showed an increased response at 10 nM. Release of IFN-γ even recovered to 60% positive cells for two donors at concentrations of 100 and 150 μM, respectively, before it significantly decreased at higher concentrations.

Treatment with CK59 also generally decreased the number of degranulating NK cells, with a reduction to 75% at 100 μM and almost complete abrogation at 150 μM (Figure 2B). At the lowest concentration of 10 μM, however, NK cells of some donors showed a slightly increased degranulation response. The effect on cytokine release was more pronounced. The number of TNF-α positive cells was reduced to about 25% at 100 μM, and diminished further at 150 μM. Strikingly, the response of two donors was not inhibited but significantly increased at low drug concentrations with a peak of 250% at 20 μM for one donor, which at higher concentrations then dropped to 20% TNF-α positive cells in a dose-dependent manner. The release of IFN-γ decreased dose-dependently in two other donors. One of those, however, showed an increased number of positive cells at 10 μM. More striking, two donors showed an increased response of 140% at 20 μM, with a decreased number of positive cells at higher concentrations. One donor even showed a pronounced increase of cells releasing IFN-γ, peaking at 50 μM with 340% positive cells compared to the corresponding control.

The effect of CID755673 on pure NK cell degranulation was moderate in comparison to PBMCs. CID755673 caused only limited but significant decrease in CD107a surface expression in all donors except one, who showed a remarkable decrease at 200 μM (Figure 2C). Two donors also showed a moderate increase of CD107a surface expression at concentrations of 50 and 100 μM, whereas the response of a further donor showed a similar response signature at concentrations of 100 and 200 μM. Higher concentrations of CID755673 consistently led to a robust decrease of TNF-α release in all donors. However, the number of TNF-α releasing cells was dramatically increased at low concentrations of CID755673 for two donors, while another one showed a small increase at 10 μM. Similar, all donors showed almost complete abrogation of IFN-γ release at concentrations of 100 μM or higher, whereas donor variations became obvious at lower concentrations. Two donors showed a pronounced increase of IFN-γ positive cells to 270 and 200%, respectively, after initial reduction at lower concentrations. Two other donors also showed a small recovery of the IFN-γ response.

In summary, all inhibitors can decrease effector functions and in a cumulative view, data obtained from pure NK resemble those obtained from PBMCs. However, we observed a high number of individual donor variations, resulting in recovering responses at higher inhibitor concentrations after an initial decrease. Additionally, some donors showed an increased response at low inhibitor concentrations, with a subsequent decrease at higher concentrations. All those variations were neither restricted to single donors, nor to inhibitors or responses.

## Discussion

Generally NK cell activation can be subdivided into two mechanisms: CD16-mediated ADCC and “natural” cytotoxicity. Whereas CD16 engagement alone is sufficient to induce prominent NK cell effector functions, natural cytotoxicity depends on the interaction between multiple activating receptors and the absence of inhibitory receptors. In a previous study it was demonstrated that engagement of CD16 as well as co-engagement of 2B4 + DNAM-1 cause responses predominantly in the same set of protein kinases, including CaMKII and PKD2, indicating their role in a core signal network. However, the existence of a core signal network, acting in both ADCC and natural cytotoxicity had to be clarified. Here, K562 tumor cells were used to activate natural cytotoxicity and to analyze the impact of the kinase inhibitors (Dasatinib, CK59, and CID755673) on NK cell effector functions. K562 tumor cells express ligands for receptors promoting natural cytotoxicity (Nectin-2/PVR for DNAM-1; MICA/B/UBLP1 for NKG2D), but do not express the 2B4 ligand CD48 or classical HLA-A, B, C, and non-classical HLA-E (Hanson et al., 2007). When comparing the signaling pathways induced by CD16-mediated ADCC- and K562-mediated natural cytotoxicity, one has to take into account the involved receptors. CD16 associates with TCRζ or/and FcRγ, initiating the classical ITAM pathway of SFK-mediated tyrosine phosphorylation, whereupon Syk association leads to PI3K, Vav1, and PLCγ-1 and 2 activation (Ting et al., 1995). This pathway leads to cytoskeletal re-arrangements through the activation of Rho, Rac, and Cdc42, and under MAPK involvement also to degranulation. K562-mediated cytotoxicity includes receptor signaling of DNAM-1/LFA-1, NKG2D, and NKp30. The three tyrosine residues of DNAM-1 can be phosphorylated by SFKs leading to the recruitment of actin-binding proteins. DNAM-1 signaling acts always co-activating, depends on its association with LFA-1 and therefore promotes cellular adhesion (Shibuya et al., 2003). NKG2D signaling shares common features with the CD16 pathway, but a DAP10 motif is associated with its receptor instead of an ITAM (CD16). Notably, NKG2D signal transduction pathways work independently of the trans-membrane adapter molecules LAT and NTAL and do not require SYK or ZAP70 kinases, instead of ADCC-mediated signaling (Billadeau et al., 2003) that mainly induces degranulation. NKp30 associates also with TCRζ and/or FcRγ chains, through the ITAM motif of the associated partner chains. In conclusion, NK cell activation is very similar to signaling events known from T or B cell activation, especially with regard to the ITAM-based NK cell receptors such as CD16 and NKp30.

Here, we investigated the effects of kinase inhibition by Dasatinib, CK59, and CID755673 on K562-induced natural cytotoxicity of NK cells within PBMC populations and pure NK cells isolated from human blood donors. They have been established as valid tools for enzyme inhibition in particular at the level of primary human immune cells (Krieg and Ullrich, 2012).

Dasatinib, formerly BMS-354825 or Sprycel^®^, is a multi-targeted kinase inhibitor that is not restricted to SFKs but also targets, e.g., the tyrosine kinase BCR-ABL1 (Lombardo et al., 2004), and is generally used as drug in leukemia therapy. Due to the structural homology of SFKs (Boggon and Eck, 2004), Dasatinib has a relatively broad target spectrum. Therefore, Dasatinib serves as a useful tool for proof-of-concept experiments, as SFKs play a major role in NK cell activation (Vivier et al., 2004; Lanier, 2008). The potency of Dasatinib was underlined by previous experiments, which showed its efficiency in inhibiting the effector functions of NK cells *in vitro* (Blake et al., 2008; Fraser et al., 2009; Salih et al., 2010). Consequently, the results of our study were completely in accordance with the data of Salih and colleagues. Additionally, our results showed remarkably reduced degranulation events and TNF-α secretion in PBMC fractions and pure NK cells. However, it was demonstrated recently that a 24-h-pretreatment with Dasatinib, followed by wash off led to dose-dependent enhancement of NK cell cytokine production, CD107a expression, and cytotoxicity against selected lymphoma and leukemia cell lines (Hassold et al., 2012). This ability of NK cells to recover effector functions after longer Dasatinib pretreatment might be explained by physiological adaptation of the cells to drug exposure. In contrast to Dasatinib, CK59, and CID755673 are considered as highly specific kinase inhibitors.

CK59 is an analog of the kinase inhibitor olomoucine, which was demonstrated to specifically inhibit CaMKII isoforms. Indeed, CaMKII-γ and CaMKII-δ were found to be the kinases with the highest affinity to immobilized CK59 (Konstantopoulos et al., 2007). Incubation of immune-precipitated CaMKII *in vitro* showed that 10 μM CK59 reduce CaMKII activity by 55%. It has already been shown that inhibition of CaMKII prevents NK cell-mediated killing of autologous dendritic cells (DC) (Poggi et al., 2002). The secretion of IFN-γ by NK cells upon contact with DCs, which activates the DCs, is also inhibited. Interestingly, our results are at variance with the results of Poggi and colleagues. Poggi and colleagues reported that the inhibition of CaMKII did not significantly reduce K562 target cell killing by NK cells, whereas this was demonstrated in this study. To explain these differences, it has to be considered that we studied degranulation and cytokine release, whereas Poggi and colleagues analyzed the specific lysis of target cells, measured by Chromium^51^ release. Furthermore, we analyzed degranulation after 2 h in CD3^−^CD56^+^ NK cells and Poggi and colleagues after 4 h in CD3^−^CD16^+^ NK cells, also using completely different CaMKII inhibitors (KN62 and KN93). Therefore, CaMKII might only be involved in cellular processes coordinating degranulation, but not the killing of target cells.

According to the PubChem database CID755673 inhibits exclusively the PKD kinase family comprising PKD1, PKD2, and PKD3 and does not exhibit any biological effect in 200 other assays (Sharlow et al., 2008). No inhibition is documented for the PKD upstream protein kinase C (PKC) or CaMKII that was also investigated here. This further underlines its high specificity, as these kinases are closely related to PKD indicated by high overall sequence homology (PKC; Sturany et al., 2001) and high homology of the catalytic domain (CAMKs; Sharlow et al., 2008), respectively. PKD2 is considered as a signaling component in NK cells but functional data are currently missing. PKD isoforms are not involved in integrin-mediated lymphocyte adhesion and homing to lymphoid tissues (Matthews et al., 2012). In the present study, application of CID755673 resulted in a significant alteration of degranulation and cytokine release. These observations suggest that PKD family kinases are participating in the signal network coordinating NK cell effector functions.

**Figure 3:**
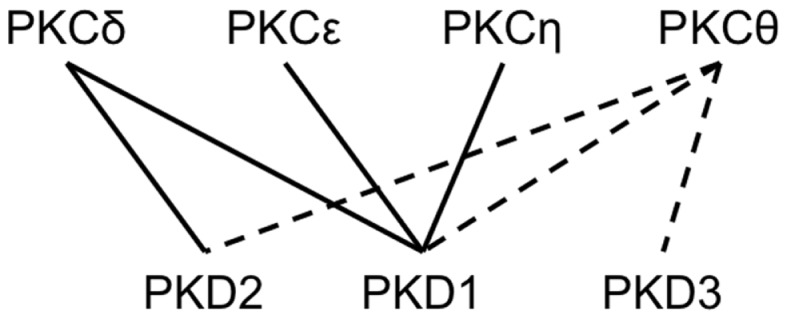
**Schematic representation of known and potential interactions between PKC and PKD family kinase isoforms**. Continuous lines indicate known interactions between specific PKC and PKD family kinase isoforms leading to activation through phosphorylation of the latter. Potential interactions of specific PKDs with PKCθ isoforms, to this date not experimentally validated, are shown as dashed lines.

Dose-dependent alterations of effector functions were also found for all three inhibitors with pure NK cells maintained by IL-2. However, in comparison to studies performed in PBMCs the caused alterations were distinct in two aspects: (i) individual donors exhibited remarkably different response profiles and (ii) all inhibitors have the potential to even increase NK effector functions. Both effects indicate that the targeted kinases can also contribute to inhibitory pathways. Protein kinases are often recognized as “overconnected” and, e.g., Fyn is part of more than 50 canonical pathways. Their pathway-specific function is assumed to be realized by distinct post-translational modifications coordinating both kinase activity and substrate binding. Previously, it was shown that the PKD2 activation status correlates directly with *in vivo* phosphorylation of S876 within transfected HEK293 cells (Sturany et al., 2001) and CaMKII activation depends on phosphorylation at Thr286 (Larsson and Broman, 2005). König and colleagues described the regulation of further modifications so far not functionally characterized. NK cell receptor engagements induce phosphorylation of PKD2 at serine 214 and of CaMKIIat serine 330 (König et al., 2012). Several other phosphorylations sites are known but a detailed mechanistic model for both kinases is still missing. However, the expression of kinase isoforms has to be taken into account to better understand donor variations and apparently contradicting results in this research field: it is known that different protein kinase C isoforms (PKCδ, ε, θ, and η) are acting as upstream signal components of PKD family kinase isoforms (PKD1, PKD2, and PKD3) (Yuan et al., 2002; Rozengurt et al., 2005; Döppler and Storz, 2007) making this signaling network even more complex. Presently it is known that PKCδ phosphorylates PKD1 and PKD2, whereas PKCε and PKCη exclusively activate PKD1 (Waldron et al., 1999; Brändlin et al., 2002; Döppler and Storz, 2007). Data concerning the specific isoforms interacting with PKCθ and PKD3 and leading to their phosphorylation are missing. Notably, PKCθ is essential for ITAM-mediated release of IFN-γ and TNF-α, but not for NK cell-mediated cytotoxicity or cytokine release induced by IL-12 and IL-18 (Tassi et al., 2008). In contrast, Page et al. (2008) determined a diminished IL-12-induced IFN-γ response of NK cells in PKCθ deletion mutant mice (PKCθ^−/−^), but did not detect a reduction of cytotoxic capacity against YAC-1 cells. Furthermore, PKCθ^−/−^ mice showed reduced, but not abrogated degranulation against MHC-I negative RMA-S tumor cells (Aguiló et al., 2009). Thus, it was shown that, chemical inhibition of the PKC downstream signaling component PKD leads to a more pronounced inhibition of NK cell effector functions than the isoform-specific knock-out of upstream kinase PKCθ. We hypothesize that the signaling pathway leading to NK cell activation depends not solely on PKCθ, but also involves other isoforms of PKC that phosphorylate one or more PKD isoforms (Figure 3).

In conclusion, chemical inhibition of either CaMKII or PKD family kinases limits NK cell effector functions. Results from natural cytotoxicity further substantiated the role of these kinases in NK cell signaling. However, the results with pure NK cells also indicate the importance to further investigate the molecular basis of donor variations, which may be caused either by differential pre-activation of kinases at the level of post-translational modifications or simply by differential expression of kinase isoforms.

## Author Contributions

Conceived and designed the experiments: Maxi Scheiter, Sebastian König, Lothar Jänsch. Performed the experiments: Maxi Scheiter, Björn Bulitta. Performed statistical evaluation: Frank Klawonn, Björn Bulitta. Analyzed the data: Björn Bulitta, Marco van Ham. Wrote the paper: Maxi Scheiter, Björn Bulitta, Sebastian König, Lothar Jänsch.

## Conflict of Interest Statement

The authors Maxi Scheiter, Björn Bulitta, Marco van Ham, Frank Klawonn, Sebastian König (during experimental work), and Lothar Jänsch declare their affiliation to Helmholtz-ZentrumfürInfektionsforschung GmbH. This does not alter the authors' adherence to all the frontiers policies on sharing data and materials.
